# Heat Transfer Enhancement During Water and Hydrocarbon Condensation on Lubricant Infused Surfaces

**DOI:** 10.1038/s41598-017-18955-x

**Published:** 2018-01-11

**Authors:** Daniel J. Preston, Zhengmao Lu, Youngsup Song, Yajing Zhao, Kyle L. Wilke, Dion S. Antao, Marcel Louis, Evelyn N. Wang

**Affiliations:** Department of Mechanical Engineering, Massachusetts Institute of Technology, Cambridge, Massachusetts 02139 USA

## Abstract

Vapor condensation is routinely used as an effective means of transferring heat or separating fluids. Dropwise condensation, where discrete droplets form on the condenser surface, offers a potential improvement in heat transfer of up to an order of magnitude compared to filmwise condensation, where a liquid film covers the surface. Low surface tension fluid condensates such as hydrocarbons pose a unique challenge since typical hydrophobic condenser coatings used to promote dropwise condensation of water often do not repel fluids with lower surface tensions. Recent work has shown that lubricant infused surfaces (LIS) can promote droplet formation of hydrocarbons. In this work, we confirm the effectiveness of LIS in promoting dropwise condensation by providing experimental measurements of heat transfer performance during hydrocarbon condensation on a LIS, which enhances heat transfer by ≈450% compared to an uncoated surface. We also explored improvement through removal of noncondensable gases and highlighted a failure mechanism whereby shedding droplets depleted the lubricant over time. Enhanced condensation heat transfer for low surface tension fluids on LIS presents the opportunity for significant energy savings in natural gas processing as well as improvements in thermal management, heating and cooling, and power generation.

## Introduction

Vapor condensation is routinely used as an effective means of transferring heat or separating fluids. Filmwise condensation is prevalent in typical industrial-scale systems, where the condensed fluid forms a thin liquid film due to the high surface energy associated with many industrial materials^1^. Conversely, dropwise condensation, where the condensate forms discrete liquid droplets which grow, coalesce, and shed, results in an improvement in heat transfer performance of an order of magnitude compared to filmwise condensation^2–5^. During water condensation, the dropwise mode is promoted with thin hydrophobic coatings^4^. However, low surface tension fluid condensates such as hydrocarbons pose a unique challenge since the typical hydrophobic condenser coatings used to shed water (surface tension *γ *≈ 73 mN/m) often do not repel fluids with lower surface tensions (*γ* < 30 mN/m). This is particularly relevant for natural gas processing applications^6^. Reentrant and doubly-reentrant surface designs have been proposed for repellency of low surface tension impinging droplets^7,8^, but these schemes are not useful during condensation when the impinging fluid can nucleate within the structures and subsequently render the surface hydrophilic^9,10^.

Meanwhile, lubricant infused surfaces (LIS) have found use in biological, lap-on-a-chip, anti-icing, and microfluidics applications, among others^11–17^. A LIS is comprised of a rough structured solid surface into which a lubricant is “infused,” or spontaneously wicked, and on which an impinging fluid ideally forms discrete droplets which easily shed from the surface. Recent work has indicated that LIS can promote formation of highly mobile droplets of low surface tension fluids, including hydrocarbons with surface tensions as low as pentane’s (*γ *≈ 16 mN/m)^18^. LIS have also been shown to improve condensation heat transfer of water in the dropwise mode^19^. The natural combination of these two research directions is the use of LIS to promote dropwise condensation of low surface tension fluids. The behaviour during condensation of hydrocarbons and other low surface tension fluids on LIS has been reported qualitatively and suggests that LIS are a promising solution to promote dropwise condensation of hydrocarbons, but no experimentally-measured improvement in heat transfer has been reported^20^.

In the present work, we quantitatively confirmed the effectiveness of LIS in promoting dropwise condensation. First, we experimentally measured condensation heat transfer coefficients during water condensation in a controlled environmental chamber in the filmwise mode and during dropwise condensation on a flat hydrophobic surface and a LIS. Then, the heat transfer performance was determined during condensation of the hydrocarbon toluene (*γ *≈ 28 mN/m) on bare and hydrophobic flat surfaces in the filmwise mode and on LIS-coated tubes in the dropwise mode at a range of supersaturations typical for natural gas processing applications. From these results, the heat transfer coefficient for hydrocarbon condensation on LIS was obtained experimentally. The ≈450% experimentally observed improvement in heat transfer for low surface tension fluids condensing on LIS presents the opportunity for significant energy savings not only in natural gas processing but also in applications such as thermal management, heating and cooling, and power generation.

## Experiment

In order to perform condensation experiments, we first fabricated tube condenser samples. The tube samples used to promote filmwise condensation of both water and toluene were bare copper (Cu) which was first solvent cleaned and then plasma cleaned. Commercially available oxygen-free Cu tubes (99.9% purity) with outer diameters *D*_*OD*_ = 6.35 mm, inner diameters *D*_*ID*_ = 3.56 mm, and lengths *L* = 131 mm were obtained. Each Cu tube was cleaned in an ultrasonic bath with acetone for 10 minutes and rinsed with ethanol, isopropanol, and deionized (DI) water. Next, the tubes were dipped into a 2.0 M hydrochloric acid solution for 10 minutes to remove the native oxide film on the surface, then triple-rinsed with DI water and dried with clean nitrogen gas (99.9%, Airgas). Finally, within 30 minutes before any experiment using the bare Cu tubes, the samples were cleaned with argon plasma to remove adsorbed hydrocarbons which are known to render metal and metal oxide surfaces hydrophobic^21–24^.

The tube sample used to promote dropwise condensation of water was functionalized with a monolayer of the hydrophobic coating octadecyltrichlorosilane (OTS), but this sample was unable to promote dropwise condensation of toluene, as discussed later. A bare copper tube cleaned as described for the hydrophilic samples above was immersed in a 0.1% by volume solution of OTS (>90%, Sigma-Aldrich) in n-hexane (99%, Sigma-Aldrich) for 5 minutes as detailed in prior work^25,26^. The coating had typical advancing/receding water contact angles of *θ*_*a*_/*θ*_*r*_ ≈ 104/93 ± 3° when measured on a flat reference surface.

The tube sample used to test condensation of both water and toluene on a LIS was a copper tube which was first coated with copper oxide (CuO) nanoblades etched following a well-known procedure^27–32^, then functionalized with a monolayer coating of trichloro(1H, 1H, 2H, 2H-perfluorooctyl)silane (TFTS) to reduce the surface energy^33,34^, and finally infused with the lubricant, Krytox GPL 101 fluorinated oil. CuO nanostructures were chosen in this study due to their suitability for copper condenser tubes; however, other options exist for fabrication of successful LIS, including silicon^20^, aluminium oxide^35^, and zinc oxide^36^ micro- and nanostructures. To create the CuO nanostructures, a bare copper tube cleaned as described for the hydrophilic samples above was immersed into a hot (96 ± 3 °C) alkaline solution composed of NaClO_2_, NaOH, Na_3_PO_4_•12H_2_O, and DI water (3.75 : 5 : 10 : 100 wt.%)^27,33^. During the oxidation process, a thin (≈300 nm) Cu_2_O layer was formed that then re-oxidized to form sharp, knife-like CuO oxide nanoblades with heights of *h* ≈ 1 μm, solid fraction *φ *≈ 0.038, and roughness factor *r *≈ 4. The CuO structures were then functionalized with TFTS (Sigma-Aldrich), which was deposited from the vapor phase. Prior to silane deposition, the tube was oxygen plasma cleaned for 2 hours to remove organic contaminants from the surface. Once clean, the tube was immediately placed in a vacuum desiccator (06514-10, Cole Parmer) with a small amount of liquid TFTS. The desiccator was evacuated by a roughing pump for 2 minutes to a minimum pressure of ≈2 kPa. A valve was then closed to isolate the pump from the desiccator and the tube was held under vacuum (≈2 kPa) for 10 minutes. The functionalized tube was then rinsed in ethanol and DI water and dried in a clean nitrogen stream (99.9%, Airgas). The TFTS coating had a typical advancing water contact angle of *θ*_*a*_ ≈ 119° when measured on a flat reference surface and typical advancing/receding water contact angles of *θ*_*a*_/*θ*_*r*_ ≈ 171/167 ± 3° when measured on the functionalized nanostructured CuO surface. The surface was infused with lubricant by first placing a droplet of Krytox GPL 101 lubricant with an approximate diameter of 2 mm onto the surface and allowing it to spread, then using a clean nitrogen stream (99.9%, Airgas) to ensure that the lubricant had spread completely. The lubricant layer thickness and thermal resistance are examined in detail in the Supplementary Information, Section S7. Advancing and receding contact angle data for both water and toluene on all of the surfaces used in the present work are presented in Table 1.

**Table 1:** Advancing and receding contact angle (degrees) reported for water and toluene on the surfaces fabricated in this work.

	Water		Toluene	
	Advancing	Receding	Advancing	Receding
Bare Copper	≈0	≈0	≈0	≈0
Flat Hydrophobic Surface	104 ± 3	93 ± 3	29 ± 3	11 ± 5
Lubricant Infused Surface	108 ± 3	105 ± 3	58 ± 3	54 ± 3
Superhydrophobic Surface	171 ± 3	167 ± 3	≈0	≈0

The superhydrophobic surface is the structured superhydrophobic CuO used to fabricate the LIS, but *without* lubricant added.

## Results

**Figure 1:**
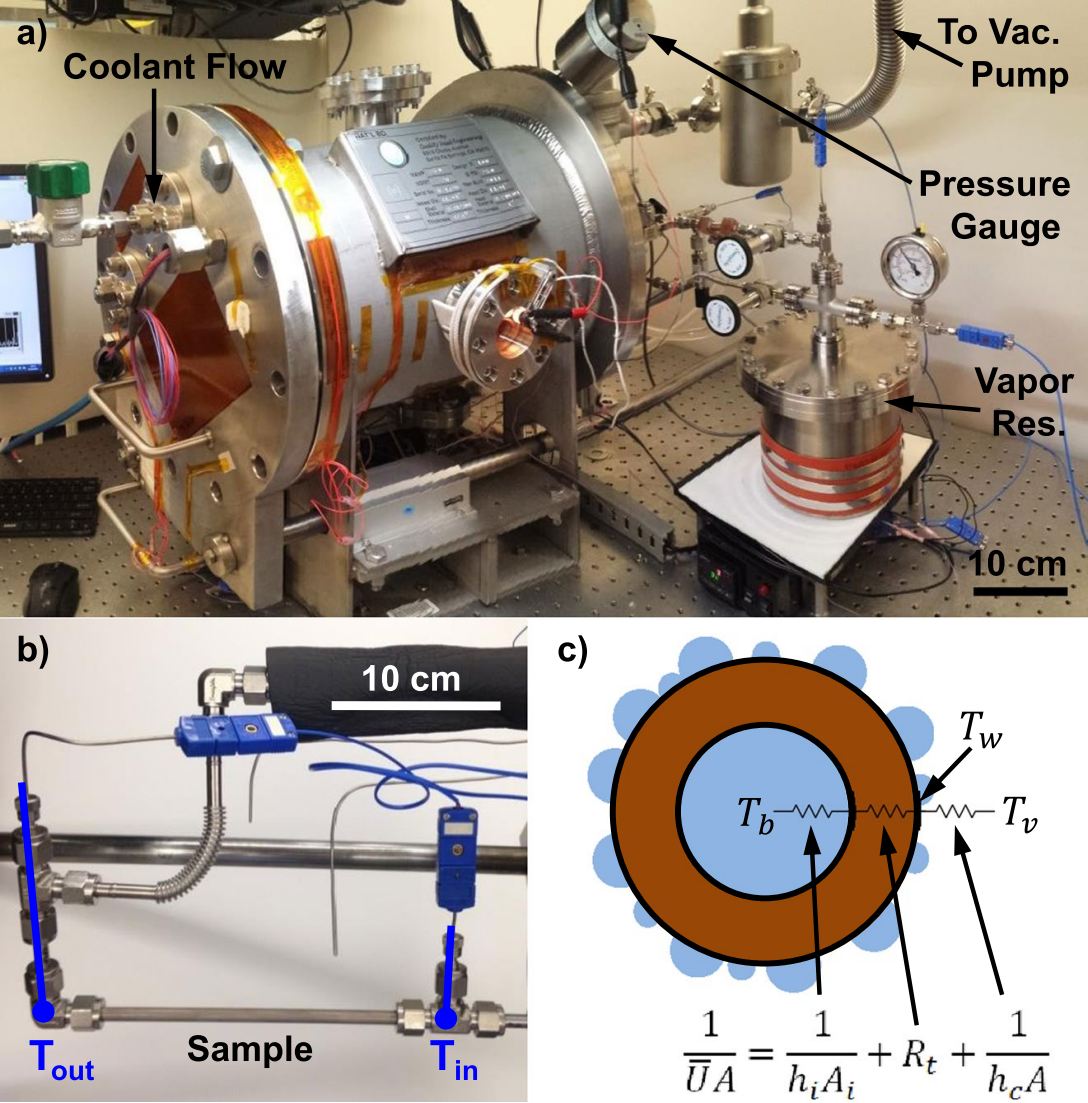
Environmental chamber with tube condenser sample to experimentally measure condensation heat transfer performance. The environmental chamber (**a**) was evacuated to <1 Pa to remove noncondensable gases. Pure, degassed vapor was introduced into the chamber from a reservoir and condensed on the exterior surface of the tube sample (**b**), while the sample temperature was maintained by a flow of coolant through the tube interior. The condensation heat transfer coefficient, *h*_*c*_, and subcooling, ∆*T* = *T*_*v*_ − *T*_*w*_, were determined from a thermal resistance network (**c**) for the tube sample.

Experiments were conducted in an environmental chamber (Fig. 1(a)). The chamber allowed the level of noncondensable gases (NCGs) to be controlled via a vacuum pump, including complete removal of NCGs from the system (<1 Pa). Following removal of NCGs, pure, degassed vapor of the condensing fluid (either water or toluene) was introduced into the chamber from a heated, temperature-controlled canister and allowed to condense on the sample. The vapor pressure was varied from 2 to 5 kPa for water and from 2.5 to 5.5 kPa for toluene in order to set the subcooling, *T*_*v*_ − *T*_*w*_, where *T*_*v*_ was measured and *T*_*w*_ was determined from the thermal resistance network shown in Fig. 1(c). Controlling for subcooling, the variation in heat transfer coefficient due to change in vapor pressure over this range is less than 5%^37^. The sample temperature was maintained with an internal flow of coolant water, where the sensible heating of the coolant fluid from the inlet to the outlet of the sample was characterized with thermocouples and used to determine the overall heat flux (Fig. 1(b)). The condensation heat transfer coefficient, *h*_*c*_, and subcooling, ∆*T* = *T*_*v*_ − *T*_*w*_, were then calculated from the thermal resistance network shown in Fig. 1(c), where the thermal resistances of the internal flow and conduction through the tube wall are known. Operation of the environmental chamber and the procedure for calculation of the condensation heat transfer coefficient, including the error analysis, are detailed in the Supplementary Information.

**Figure 2:**
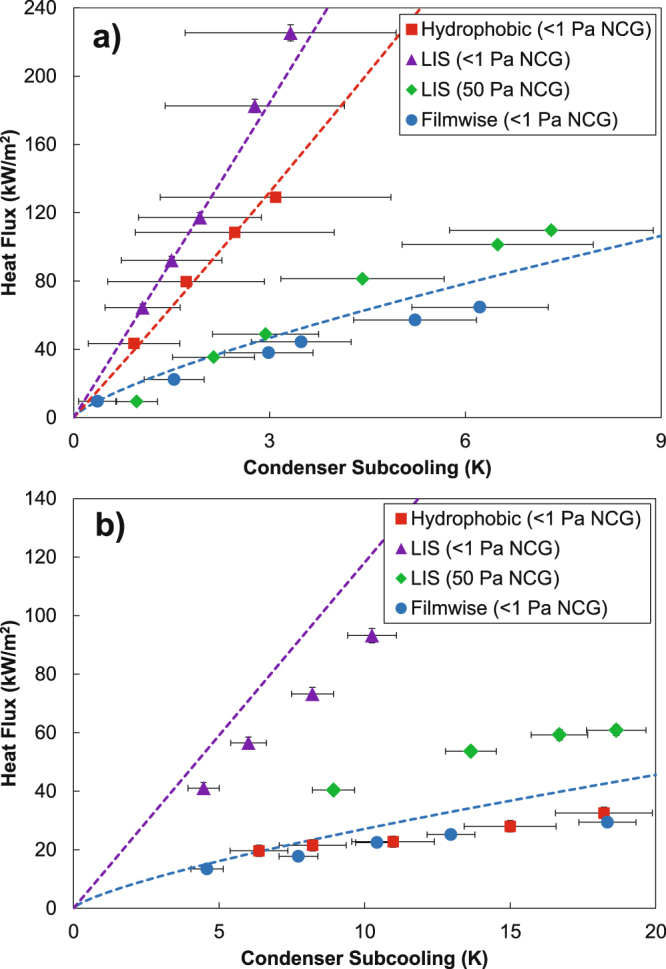
Heat flux as a function of condenser subcooling (∆*T* = *T*_*v*_ − *T*_*w*_) for water and toluene, with experimental results as points and model predictions as dashed lines. (**a**) Water is condensed onto a bare copper tube in the filmwise mode, a flat hydrophobic copper tube in the dropwise mode, and a LIS-coated copper tube in the dropwise mode in pure vapor and with 50 Pa of noncondensable gas (NCG) present in the chamber. (**b**) Toluene is condensed onto a bare copper tube in the filmwise mode and a LIS-coated copper tube in the dropwise mode in pure vapor and with 50 Pa of NCG present in the chamber. Toluene condensation on the flat hydrophobic copper tube resulted in the filmwise mode, evidenced by the agreement between the experimental data for this case and the model for filmwise condensation.

We first characterized filmwise condensation of both water and toluene on the bare copper tubes and compared the results to Nusselt’s falling-film theory in order to validate the experimental results^1^. The experimental results were in good agreement with Nusselt’s model for both water and toluene (Fig. 2(a,b)). The slight overprediction by the model (dotted lines) is attributed to the assumption that fluid reaching the bottom of the tube is immediately removed, while in reality the fluid accumulates at the bottom of the tube and eventually sheds as droplets, resulting in a higher average conduction resistance through the condensing fluid than in Nusselt’s model.

**Figure 3:**
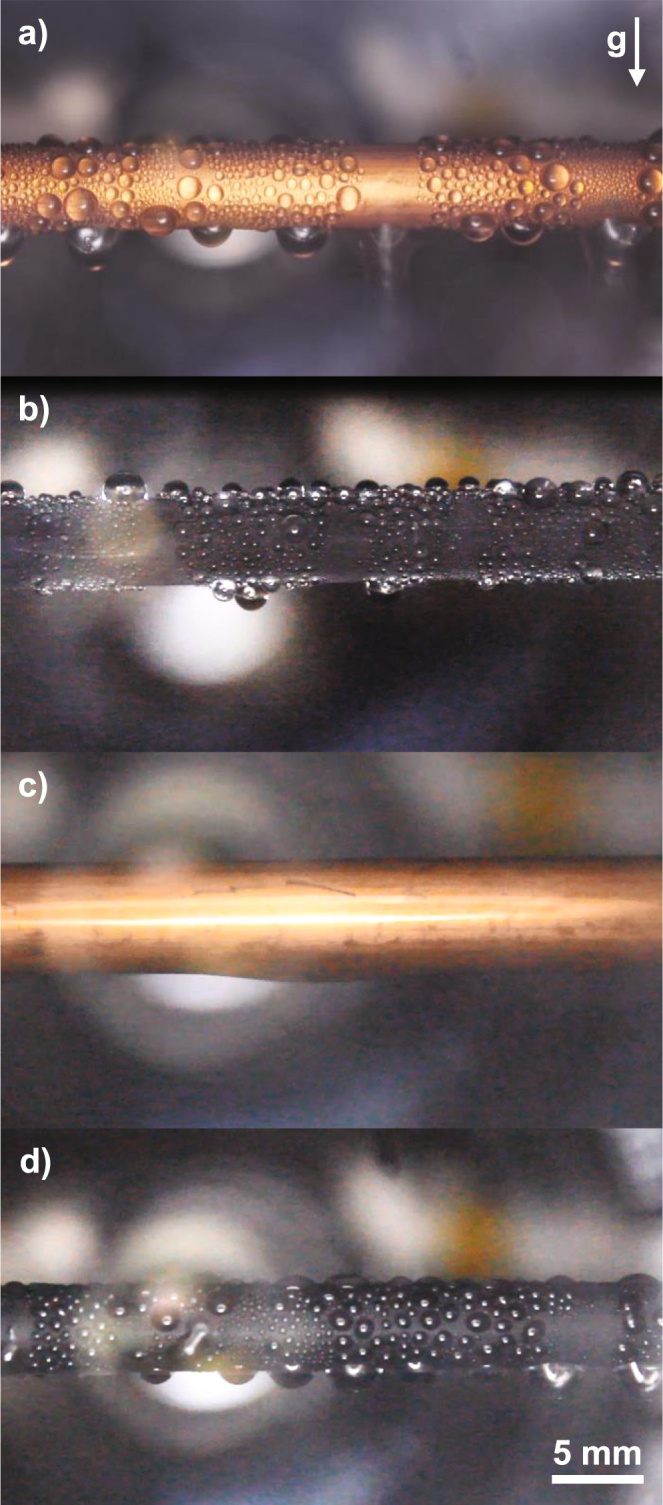
Images of condensation of water (**a**,**b**) and toluene (**c**,**d**). Water is condensed on the flat hydrophobic surface in (**a**) and on the LIS in (**b**). Toluene is condensed on the flat hydrophobic surface in (**c**) and on the LIS in (**d**). Droplet departure diameters were calculated from videos of condensation and used in the model to predict the expected dropwise heat transfer coefficients on the flat hydrophobic surface and the LIS (see Supplementary Information).

We went on to characterize the condensation of water and toluene on the tube with a flat hydrophobic coating, where we observed that water underwent dropwise condensation (Fig. 3(a)) but toluene exhibited filmwise behaviour (Fig. 3(c)). While we initially observed the nucleation and growth of small, discrete droplets of toluene on the flat hydrophobic surface, at any appreciable heat flux, the toluene transitioned to filmwise condensation as can be expected for a condensate with low contact angle and non-negligible contact angle hysteresis, shown in detail in Figure S3 in the Supplementary Information^4,38,39^. This illustrates the difficulty of condensing low surface tension fluids on typical hydrophobic coatings. Meanwhile, the heat transfer performance for dropwise condensation of water outperformed filmwise condensation and was also in good agreement with a model based on individual droplet heat transfer integrated over a known droplet size distribution as shown in Fig. 2(a) (see Supplementary Information, Section S6 for model description)^40^.

Finally, we explored condensation of water and toluene on the LIS (Fig. 2). In a previous experiment in which water was condensed on a LIS performed by Xiao *et al*., the condensation heat transfer coefficient was measured experimentally and reported to be 100% greater than that of dropwise condensation on a flat surface; however, the heat transfer coefficients reported in this work for dropwise condensation on both the flat hydrophobic surface and LIS were worse than the expected value for filmwise condensation calculated from Nusselt’s model (see Supplementary Section S1)^19^. This study had included NCGs (30 Pa) in the chamber during the condensation heat transfer measurements, which are known to degrade heat transfer performance due to buildup of noncondensable gases at the condenser surface and an accompanying resistance due to vapor diffusion through the NCG layer^3,4,41^. While the NCGs were reported to serve the purpose of preventing evaporation of the lubricant^19^, we found that the vapor pressure of Krytox GPL 101 is much lower than 1 Pa, and therefore we were able to run experiments with virtually no NCG while still maintaining the presence of the Krytox lubricant on the surface. However, experiments were performed both without (i.e., <1 Pa) NCG and with 50 Pa NCG present to explore the effect of NCG on heat transfer performance and determine whether this could be the mechanism whereby Xiao *et al*. reported condensation heat transfer coefficients much lower than expected from modelling.

Condensation of both water and toluene on the LIS exhibited dropwise behaviour (Fig. 3(b,d)). In the presence of NCG, the heat transfer performance was only marginally better than filmwise condensation, rationalizing the result obtained by Xiao *et al*.^19^. When NCG was removed from the chamber, the heat transfer performance during water condensation exceeded that of dropwise condensation by ≈30% and filmwise condensation by ≈400%, as shown in Fig. 2 (note that the overlap in error bars for the experimental data points corresponding to dropwise condensation of water on the flat hydrophobic coating and the LIS does not indicate uncertainty that the flat coating may be outperforming the LIS, but rather that systematic experimental uncertainty may shift both sets of measurements in the same direction within the error bars). Meanwhile, toluene condensation on the LIS outperformed filmwise condensation by ≈450%, in good agreement with the prediction by Rykaczewski *et al*. of a ≈600% enhancement for toluene condensing on a LIS based on an approximation considering a partial droplet size distribution^20^. Furthermore, experimental results for both water and toluene condensation on the LIS were in good agreement with the condensation heat transfer model accounting for a distribution of droplet sizes, where the droplet size distribution used in the model for LIS was adjusted according to recent work (see Supplementary Information Section S6)^42,43^.

**Figure 4:**
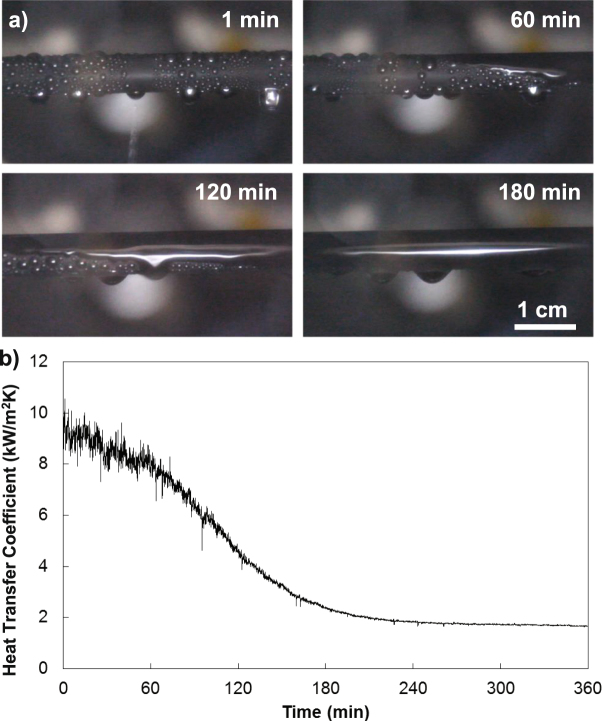
Toluene condensation on the LIS over time. Toluene initially exhibits dropwise condensation on the LIS, but within 1 hour the surface begins to transition to filmwise condensation, shown as a time-lapse sequence of images in (**a**). The lubricant was forced to the bottom of the condenser by shear force imparted by shedding droplets rendering the top of the condenser surface wettable by toluene. Correspondingly, the heat transfer coefficient degraded by approximately 78%, shown in (**b**). Upon rewetting the surface with lubricant, the surface could again shed discrete droplets of toluene, indicating that the failure was due to lubricant depletion and not structural damage.

The long-term performance of surface coatings is often a consideration when they are proposed for industrial applications. LIS are particularly concerning in this regard, as the lubricant may be depleted from the surface over time due to several mechanisms. If the droplets of condensate are “cloaked,” or covered in a thin layer of lubricant, they will carry lubricant with them during shedding and deplete the lubricant over time^44–46^. Another depletion mechanism is shearing of the lubricant, which may also occur due to droplet shedding as droplets slide over the LIS, causing accumulation of lubricant at the bottom of the condenser^47,48^. In order to test the failure mechanism of the LIS during hydrocarbon condensation, we continuously condensed toluene on the LIS over a time period of 6 hours. We found that the timescale for surface failure was on the order of 1 hour, evidenced by the time-lapse sequence of images in Fig. 4(a) and in agreement with another recent study on LIS which reported that low viscosity lubricants failed in less than 1 hour but did not explore the failure in further detail^42^. The condensation transitioned from dropwise to filmwise, with a corresponding decrease in heat transfer coefficient of ≈78% (=1–1/450%) shown in Fig. 4b. We also observed that the degradation began at the top of the condenser surface and slowly moved downwards. Since toluene is not cloaked by Krytox^20,49^, the droplet shearing effect^47,48^ is primarily responsible for the LIS failure in this case as evidenced by the accumulation of lubricant at the base of the condenser over time.

## Discussion

Applying LIS to a condenser is shown here to be a viable approach to promote dropwise hydrocarbon condensation and improve the condensation heat transfer coefficient. This is not the only solution to improve heat transfer during condensation of low surface tension fluids; in some cases, it is also possible to modify the functionalizations on flat condenser surfaces to lower the surface energy until droplet formation is energetically favorable^20^, particularly with fluorinated carbon chains^50^. However, geometric and chemical defects on solid surfaces result in contact angle hysteresis^51,52^, and a high level of contact angle hysteresis can cause a transition to filmwise condensation as heat flux increases^4^. Even traditional superhydrophobic surfaces are often unable to repel low surface tension fluids, as evidenced by the complete spreading of toluene over superhydrophobic CuO indicated in Table 1. The low contact angle hysteresis found on LIS therefore provides an advantage compared to flat or micro and nanostructured surfaces as it may allow continued shedding of droplets of low surface tension fluids at higher heat fluxes^4,18,53^.

LIS may prove more effective than flat or micro and nanostructured coatings at enhancing low surface tension fluid condensation heat transfer, but failure by depletion of the lubricant remains a critical concern. The lubricant can be depleted by departure of cloaked droplets, or, as observed in the present work, the lubricant can be depleted even in the absence of droplet cloaking due to shearing by sliding droplets. A potential solution to lubricant shearing could be the addition of barriers for lubricant flow as proposed by Wexler *et al*. in the context of fluid flow past a LIS^47,48^, or alternatively a suitable design of the solid structures on the surfaces to tune the capillary pressure and permeability governing lubricant return after shearing, which could draw from concepts proposed in literature on evaporation from wicking materials^54,55^. If justifiable in a given application, the lubricant could be replenished periodically as well to overcome the problem. The importance of NCG in condensation was also demonstrated in the present work, where less than 10% NCG was shown to eliminate the gain in performance obtained from promotion of dropwise condensation. This confirms previous results indicating the importance of even low levels of NCG on condensation performance^41,56^. Future experiments in this field should be carefully conducted in pure vapor to allow direct comparison between studies unless the target application requires NCG, such as in fog harvesting^16^.

Even in light of the challenges highlighted above that must be addressed before LIS will find practical use as a condenser coating, the enhancements in heat transfer coefficient versus filmwise condensation of 400% and 450% for water and toluene, respectively, suggest that LIS merit further exploration. Specifically, promotion of dropwise condensation of low surface tension fluids on LIS where flat coatings may not suffice due to contact angle hysteresis is a promising future direction. The demonstrated condensation heat transfer enhancement indicates more efficient natural gas processing as well as improved device thermal management, heating and cooling, and power generation are possible.

### Data availability

All data generated or analysed during this study are included in this published article (and its Supplementary Information file).

## Electronic supplementary material

Supplementary Information
