# Supplementary material for: Heat Transfer Enhancement During Water and Hydrocarbon Condensation on Lubricant Infused Surfaces
Source: Sci Rep. 2018 Jan 11;8:540. doi: 10.1038/s41598-017-18955-x (PMC5765152; doi:10.1038/s41598-017-18955-x)
Supplement: Supplementary file 1 — Supplementary Information [file 41598_2017_18955_MOESM1_ESM.pdf]

Supplementary Information for:

Heat Transfer Enhancement During Water and Hydrocarbon Condensation on Lubricant Infused Surfaces

Daniel J. Preston, Zhengmao Lu, Youngsup Song, Yajing Zhao,

*Kyle L. Wilke, Dion S. Antao, Marcel Louis, Evelyn N. Wang**

Department of Mechanical Engineering, Massachusetts Institute of Technology,

Cambridge, Massachusetts 02139, USA

**Corresponding author email: enwang@mit.edu*

S1. Results from Xiao et al. Compared to Filmwise Condensation from Nusselt's Model

Xiao et al. reported that *"the overall heat transfer coefficients on [dropwise condensation] surfaces in this work (h < 2–7 kW/m^{2}K) are much lower compared to pure vapor conditions [in previous work by Miljkovic et al.] (h < 12–13 kW/m^{2}K)[1] due to the presence of NCGs acting as a diffusion barrier to the transport of water vapor towards the condensing surface."*[2] Therefore, the fact that the noncondensables (NCGs) would degrade heat transfer performance was recognized. Unfortunately, the data reported is the overall heat transfer coefficient including the tube wall and the chiller water flow, as opposed to the isolated condensation heat transfer coefficient. We can approximate the condensation heat transfer coefficient (including NCG effects) by assuming that the effective heat transfer coefficient of the non-condensing sections of the resistance network, h_{fixed}, namely, the tube wall and the chiller water flow, remains fixed between experiments (see Figure 1 in the main text for a schematic of the resistance network):

$$h_{fixed} = \left(\frac{1}{h_{overall}} - \frac{1}{h_{cond}}\right)^{-1} = \left(\frac{1}{12\ \mathrm{kW/m^2K}} - \frac{1}{60\ \mathrm{kW/m^2K}}\right)^{-1} = 15\ \mathrm{kW/m^2K} \qquad \text{(S1)}$$

where $h_{overall}$ is taken from the earlier work by Miljkovic et al. using the same experimental setup to characterize dropwise condensation and h_{cond} is the heat transfer coefficient of dropwise condensation reported in that work.[1] Now we compare h_{fixed} with the range of overall heat transfer coefficients reported by Xiao et al., 2–7 kW/m^{2}K, and we see that the thermal resistance of the condensation with NCGs is the dominant resistance in the network in this scenario:

$$\frac{R_{cond}}{R_{overall}} = \frac{h_{overall}}{h_{cond}} = \frac{h_{overall}}{\left(\frac{1}{h_{overall}} - \frac{1}{h_{fixed}}\right)^{-1}} = 53\%\ to\ 87\% \qquad \text{(S2)}$$

Rearranging this equation, we can determine the h_{cond} including the effect of NCG for any given $h_{overall}$ reported in Figure 4 of Xiao et al. using Equation S3 here:

$$h_{cond} = \left(\frac{1}{h_{overall}} - \frac{1}{h_{fixed}}\right)^{-1} \tag{S3}$$

Next, assuming a constant wall temperature, T_w, across all experiments of 17 °C, determined from the supersaturation of 1.6 at the maximum vapor pressure plotted in Xiao et al.'s Figure 4, we can also determine the vapor temperature, T_v, at each vapor pressure for a saturated mixture,

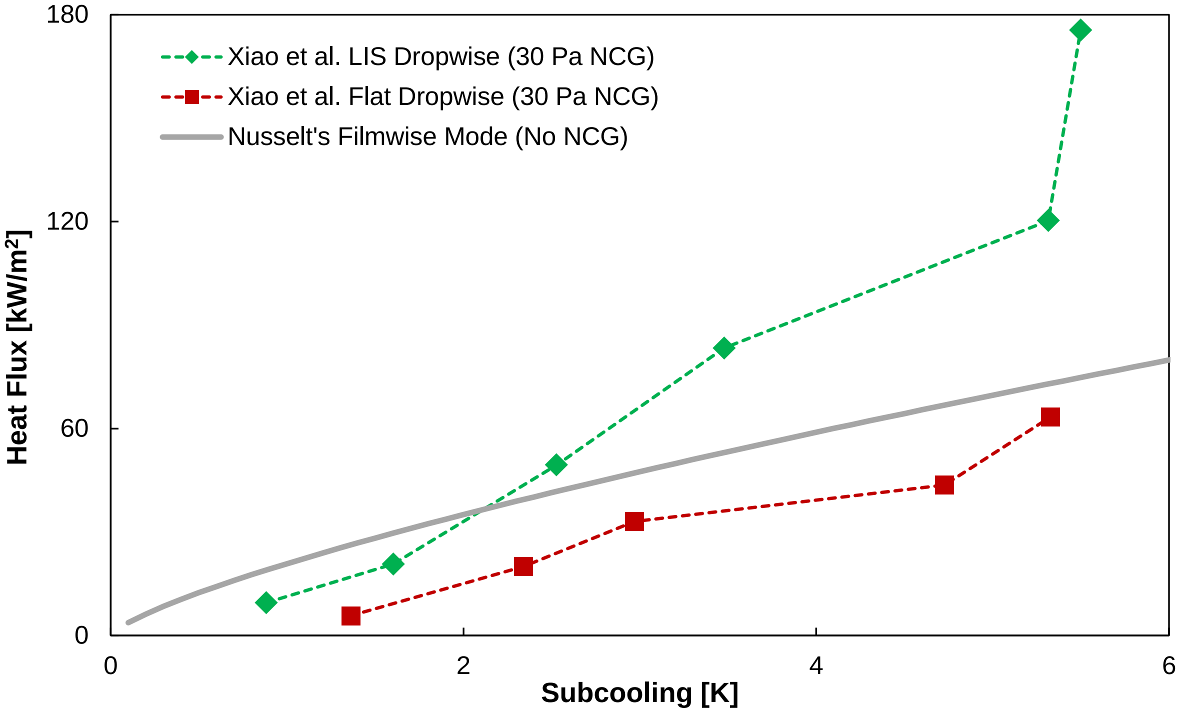

Figure S1. *Adjusted and replotted data from Xiao et al. shows heat flux vs. condenser subcooling. The 30 Pa NCG in the system degrades the heat transfer performance of the dropwise condensation to approximately that of the expectation for filmwise condensation.*

and from these two temperatures the subcooling is known. The subcooling and the condensation heat transfer coefficient can be combined to determine the heat flux for each data point. Finally, plotting the heat flux versus the subcooling for each data point yields the result shown in Figure S1, where the heat transfer is comparable to that which would be expected from filmwise condensation.

S2. Droplet Departure Size Characterization

The average droplet departure size was calculated using imaging analysis performed on videos of condensation on the different surfaces with at least 20 departure events observed for each sample. The results are shown in Table S1.

Table S1. Average droplet departure diameters.

	Average Diameter (mm)	Standard Deviation (mm)
Water on Flat Hydrophobic Surface	2.92	0.42
Water on LIS	1.23	0.34
Toluene on LIS	2.12	0.23

S3. Condensation Chamber Setup

The custom environmental chamber used for this work consists of a stainless steel frame with a door (sealed with a rubber gasket), two viewing windows, and apertures for various components. Resistive heater lines were wrapped around the exterior of the chamber walls to prevent condensation at the inside walls and then insulated on the exterior walls. The output power of the

resistive heater lines was controlled by a voltage regulator. Two insulated stainless steel water flow lines (Swagelok) were fed into the chamber to supply cooling water to the chamber from a large capacity chiller.

A secondary stainless steel tube line was fed into the chamber that served as the flow line for the incoming vapor supplied from a heated steel reservoir. The vapor line was wrapped with a rope heater (60 W, Omega) and controlled by a power supply. The vapor reservoir was wrapped with another independently-controlled heater (120 W, Omega) and insulated to limit heat losses to the environment. The access tubes were welded to the vapor reservoir, each with independently-controlled valves. The first valve (Diaphragm Type, Swagelok), connecting the bottom of the reservoir to the ambient, was used to fill the reservoir with the condensing fluid. The second valve (BK-60, Swagelok), connecting the top of the reservoir to the inside of the chamber, provided a path for vapor inflow. K-type thermocouples were located along the length of the vapor reservoir to monitor temperature.

A bellows valve (Kurt J. Lesker) was attached to the chamber to serve as a leak port between the ambient and inside of the chamber. In order to monitor temperatures within the chamber, T-type thermocouple bundles were connected through the chamber apertures *via* a thermocouple feed through (Kurt J. Lesker). A pressure transducer (925 Micro Pirani, MKS) was attached to monitor pressure within the chamber. The thermocouple bundles and the pressure transducer were both electrically connected to an analog input source (RAQ DAQ, National Instruments), which was interfaced to a computer for data recording. A second bellows valve (Kurt J. Lesker) was integrated onto the chamber for the vacuum pump, which brought down the chamber to

vacuum conditions prior to vapor filling. A liquid nitrogen cold trap was incorporated along the line from the chamber to the vacuum which served to remove any moisture from the pump-down process and ultimately assisted in yielding higher quality vacuum conditions. A tertiary bellows valve (Kurt J. Lesker) was integrated on a T fitting between the vacuum pump and liquid nitrogen reservoir to connect the vacuum line to the ambient to release the vacuum line to ambient conditions once pump-down was achieved. In order to visually capture data, a digital SLR camera (Canon EOS 50D) was placed in line with the viewing window on the chamber.

The setup used to run experiments inside the chamber is shown in Figure 1(a) in the main text. Stainless steel tube lines (1/4”, Swagelok) were connected to the external water flow lines (Figure 1(b) in the main text). T-connection adapters (Swagelok) with bore through Ultra-Torr fittings (Swagelok) were used to adapt T-type thermocouple probes (Omega) at the water inlet and outlet.

S4. Condensation Procedure

For each experimental trial, a set of strict procedures was followed to ensure consistency throughout the experiments. The first step of the process was to turn on the voltage regulator to heat up the environmental chamber walls, which prevented condensation on the chamber walls. Simultaneously, the vapor reservoir was filled with approximately 2 liters of either DI water or toluene. After opening the vapor inflow valve and closing the vapor release valve, the rope heater around the vapor reservoir was turned on with the heater controller set to maximum output (1200 W). Then the rope heater connected to the vapor inflow valve was turned on. The temperature of the reservoir was monitored with the installed thermocouples; the temperature at

the top of the reservoir was higher than that of the middle/bottom of the reservoir due to the water thermal mass present at the middle/bottom section. Hence, we ensured that the regions of the reservoir of higher thermal capacity were brought to a sufficiently high temperature for boiling. During the boiling process, aluminum foil was placed on the bottom surface of the inner chamber to collect any of the liquid leaving the vapor inflow line. Once boiling was achieved and the internal thermocouple on the reservoir was 5˚C above the boiling point for at least 10 minutes, the vapor inflow valve was closed. The excess fluid that spilled inside the chamber during de-gassing of the reservoir was removed.

In order to install the samples onto the rig, the Swagelok female adapters at the ends of the tube samples were connected to the male connecters on the rig. Before installing the entire sample setup in the chamber, all adapters/connecters were tightened to ensure that there were no leaks that could affect vacuum performance. Finally, the bellows tubes (for the chiller water inflow/outflow) were connected to the chiller water lines.

The next step was to begin the vacuum pump-down procedure. Initially, the liquid nitrogen cold trap was filled to about half capacity. The ambient exposed valves connecting the chamber and the vacuum pump were both closed and the valve connected to the liquid nitrogen cold trap was opened. The vacuum pump was then turned on, initiating the pump-down process. The pressure inside the chamber was monitored during the pump-down process. This process took approximately one hour in order to achieve the target vacuum conditions (0.5 Pa $< P <$ 1 Pa). The experimental operating pressure of non-condensable was set to be a maximum of 0.25% of the operating pressure. Non-condensable gas content of above 0.5% (pressure) has been shown

to significantly degrade performance during dropwise condensation.[3] In our experiments, extreme care was taken to properly de-gas the vacuum chamber and vapor reservoir prior to experimental testing. In addition, the chamber leak rate was characterized prior to each run in order to estimate the maximum time available for acquiring high fidelity data with non-condensable content of less than 0.25%.

The setup of the chiller water flow-loop is described as follows: the water pump reservoir was filled and turned on to a flow rate of 5 L/min. The flow rate was monitored with the flow meter integrated in the inflow water line. In order to bring the chilled water into the flow loop and to the tube sample, the external chilled water lines were opened.

Prior to beginning experiments, the camera was turned on for visual imaging of the sample during condensation. Afterwards, the rope heater around the water reservoir was turned off and the vapor inflow valve was slowly turned open until the operating pressure was reached. Steady state conditions were typically reached after 2 minutes of full operation.

S5. Heat Transfer Coefficient and Error Propagation

An energy balance was applied to the tube sample to determine the overall condensation heat transfer by calculating the change in enthalpy of the chiller water flowing inside the tube:

$$Q = \dot{m} c_p (T_{out} - T_{in}) \tag{S4}$$

where Q is the overall condensation heat transfer rate, $\dot{m}$ is the chiller water mass flow rate, c_p is the chiller water specific heat, and T_{in} and T_{out} are the tube condenser inlet and outlet temperatures, respectively. From the overall heat transfer rate, we calculated the heat flux by dividing by the condenser surface area:

$$q" = Q/A = \dot{m}c_p(T_{out} - T_{in})/A \tag{S5}$$

where A is the surface area of the outer tube surface ($A = 2\pi rL$, where r = 3.175 mm, L = 13.1 cm). The temperature difference between the chiller water and vapor far from the tube sample was also determined, represented here as the log mean temperature difference (LMTD) to account for the change in temperature of the chiller water along the tube length:

$$\Delta T_{LMTD} = \frac{(T_v - T_{in}) - (T_v - T_{out})}{\ln\left(\frac{T_v - T_{in}}{T_v - T_{out}}\right)} \tag{S6}$$

where T_v is the temperature of the surrounding vapor far from the tube sample ($T_v = T_{sat}(P_v)$). From the overall condensation heat transfer and the log mean temperature difference, the overall heat transfer coefficient, $\overline{U}$, was determined:

$$\overline{U} = \frac{Q}{A\Delta T_{LMTD}} = \frac{\dot{m}c_p(T_{out} - T_{in})}{A\Delta T_{LMTD}} \tag{S7}$$

where A is the surface area of the outer tube surface ($A = 2\pi rL$, where r = 3.175 mm, L = 13.1 cm). Note that the overall heat transfer coefficient is a function of only the product of experimentally measured parameters raised to powers. Therefore, the error associated with $\overline{U}$ is calculated as follows:

$$E_{\overline{U}} = \overline{U}\sqrt{\left(\frac{E_{\dot{m}}}{\dot{m}}\right)^2 + \left(\frac{E_{(T_{out}-T_{in})}}{(T_{out} - T_{in})}\right)^2 + \left(\frac{-E_A}{A}\right)^2 + \left(\frac{-E_{\Delta T_{LMTD}}}{\Delta T_{LMTD}}\right)^2} \tag{S8}$$

The condensation heat transfer coefficient can be extracted from $\overline{U}$ by considering a series of thermal resistances that sum to $\overline{U}$ and isolating the resistance associated with condensation:

$$\frac{1}{\overline{U}A} = \frac{1}{h_i A_i} + R_t + \frac{1}{h_c A} \tag{S9}$$

Rearranging to solve explicitly for h_c:

$$h_c = \left(\frac{1}{\overline{U}} - \frac{A}{h_i A_i} - R_t A\right)^{-1} \tag{S10}$$

where A_i is the surface area on the inner surface of the tube ($A_i = 2\pi r_i L$), R_t is the thermal resistance of the tube ($R_t = \ln(r/r_i)/(2\pi k_t)$, k_t is the tube material thermal conductivity), and the internal heat transfer coefficient, h_i, is determined from the Gnielinski correlation for pipe flow:

$$h_i = \left(\frac{k_i}{2r_i}\right)\frac{(f/8)(Re - 1000)Pr}{1 + 12.7(f/8)^{1/2}(Pr^{2/3} - 1)} \tag{S11}$$

$$f = (0.790 \ln Re - 1.64)^{-2} \tag{S12}$$

$$Re = \frac{\rho v(2r_i)}{\mu} \tag{S13}$$

where f is the friction factor, Re is the Reynolds number, Pr is the Prandtl number, ρ is the chiller water density, k_i is the chiller water thermal conductivity, and μ is the chiller water dynamic viscosity. Solving for h_i and substituting into the above Equation S10 allows for determination of h_c. Once h_c is known, the condenser surface subcooling is determined as follows:

$$\Delta T = T_v - T_w = \frac{q"}{h_c} \tag{S14}$$

As h_c is not a simple function of a product of powers, the error is determined as a function of the first partial derivatives of h_c with respect to its components.

$$E_{h_c} = h_c\sqrt{\left(\frac{\partial h_c}{\partial h_i}E_{h,i}\right)^2 + \left(\frac{\partial h_c}{\partial \overline{U}}\frac{E_{\overline{U}}}{\overline{U}}\right)^2} \tag{S15}$$

$$\frac{\partial h_c}{\partial h_i} = \frac{-(A/A_i)\overline{U}^2}{(h_i - (A/A_i)\overline{U} - R_t A\overline{U}h_i)^2} \tag{S16}$$

$$\frac{\partial h_c}{\partial \bar{U}} = \frac{{h_i}^2}{(h_i - (A/A_i)\bar{U} - R_t A \bar{U} h_i)^2} \tag{S17}$$

The error in $\bar{U}$ was determined in Equation S8 and the error in h_i was estimated as 10% associated with the Gnielinski correlation.[4] Table S2 below summarizes the uncertainty associated with each experimental measurement.

Table S2. Uncertainties corresponding to experimental measurements.

**Experimental Measurement**	**Uncertainty**
Chiller water temperature difference $(T_{out} - T_{in})$	0.1K
Saturated vapor pressure (P_v)	1%
Saturated vapor temperature (T_v)	$T_{sat}(1.01(P_v))$-$T_{sat}(P_v)$
Chiller water mass flow rate ($\dot{m}$)	2%
Sample surface area (A)	2%
Gnielinski correlation heat transfer coefficient (h_i)	10%

S6. Modeling of Heat Transfer Coefficient

To model dropwise condensation, $h_{c,d}$ was obtained by incorporating the individual droplet heat transfer with droplet size distribution:[5]

$$h_{c,d} = \frac{q"}{\Delta T} = \frac{1}{\Delta T}\left(\int_{R^*}^{R_e} q(R)n(R)dR + \int_{R_e}^{\hat{R}} q(R)N(R)dR\right) \tag{S18}$$

$$q(R) = \frac{\pi R^2\left(\Delta T - \frac{2T_{sat}\sigma}{Rh_{fg}\rho_w}\right)}{\frac{1}{2h_{int}(1-\cos\theta)} + \frac{R\theta}{4k_w\sin\theta} + \frac{1}{k_{HC}\sin^2\theta}\left(\frac{k_P\varphi}{\delta_{HC}k_P + hk_{HC}} + \frac{k_W(1-\varphi)}{\delta_{HC}k_w + hk_{HC}}\right)^{-1}} \tag{S19}$$

where q'' is the steady state dropwise condensation heat transfer rate per unit area of the condensing surface, ΔT is the temperature difference between the saturated vapor and sample outer surface ($\Delta T = (T_{sat}(P) - T_s)$), R^* is the critical radius for heterogeneous nucleation ($R^* = r_c$),[6] R_c is the droplet coalescence radius, $q(R)$ is the individual droplet heat transfer (Equation S19), $n(R)$ is the non-interacting droplet size distribution,[5] $N(R)$ is the coalescence dominated droplet size distribution,[5, 7] R is the droplet radius, σ is the condensate surface tension, h_{fg} is the latent heat of phase change, ρ_w is the condensate density (liquid water), θ is the droplet contact angle, h_{int} is the interfacial heat transfer coefficient,[8] k_w is the condensate thermal conductivity, k_{HC} is the hydrophobic coating thermal conductivity, φ is the structured surface solid fraction (equal to one for the flat surfaces considered here), h is the structured surface height (equal to zero for flat surfaces), and δ_{HC} is the hydrophobic coating thickness (≈1 nm).[6] The first integral in Equation S14 represents the heat flux component from droplets smaller than the coalescence

length scale ($R < R_e$), where direct growth by vapor accommodation at the liquid-vapor interface dominates and neighboring droplet coalescence is absent. The second integral represents the component of the heat flux from droplets growing mainly by coalescence with other droplets ($R > R_e$). These two components contribute to the total surface heat transfer per unit area (q''). The model results were obtained using experimentally determined droplet departure radii $\hat{R}$ (see Table S1, above) and contact angles.

Note that the equation above is modified on the lubricant infused surface because lubricant is between the structures on the surface. The appropriate equation for the LIS is:

$$q(R) = \frac{\pi R^2 \left(\Delta T - \frac{2T_{sat}\sigma}{R h_{fg}\rho_w}\right)}{\frac{1}{2h_{int}(1-\cos\theta)} + \frac{R\theta}{4k_w \sin\theta} + \frac{1}{k_{HC}\sin^2\theta}\left(\frac{k_P\varphi}{\delta_{HC}k_P + hk_{HC}} + \frac{k_W(1-\varphi)}{\delta_{HC}k_l + hk_{HC}}\right)^{-1}} \quad \text{(S20)}$$

where the k_w has been changed to k_l in the right-most section of the denominator. Another important modification to the model for LIS is the alteration of the droplet size distribution for large drops, N(R). In previous work on dropwise condensation on flat surfaces, and for the model in the present work used for dropwise condensation on a flat surface, the droplet size distribution has been taken as:

$$N(R) = \frac{1}{3\pi R^2 \hat{R}}\left(\frac{R}{\hat{R}}\right)^{-2/3} \quad \text{(S21)}$$

However, recent work by Weisensee et al.[9] has suggested that LIS follow a large droplet size distribution of:

$$N(R) = \frac{1}{3\pi R^3} \tag{S22}$$

based on experimental observation. Therefore, N(R) presented in Equation S22 above is used when modeling LIS. Finally, the droplet radius at which coalescence-based growth begins to dominate was found to vary between samples. Values of 1 μm, 4 μm, and 8 μm were used for water condensation on the flat hydrophobic surface, water condensation on the LIS, and toluene condensation on the LIS, respectively; all of these values lie in the range of typically observed droplet radii for coalescence dominated growth of 0.5–10 μm[9] and may have implications in the nucleation behaviour of droplets on LIS.

To model filmwise condensation on the smooth Cu tubes, the Nusselt model was used:[4, 8]

$$h_{c,f} = 0.729\left(\frac{g\rho_w(\rho_w - \rho_v){k_w}^3 h'_{fg}}{\mu_w(2r)\Delta T}\right)^{1/4} \tag{S23}$$

$$h'_{fg} = h_{fg} + 0.68c_{p,l}\Delta T \tag{S24}$$

where g is the gravitational acceleration (g = 9.81 m/s^2), ρ_v is the water vapor density, μ_w is the condensate dynamic viscosity, h'_{fg} is the modified latent heat of vaporization accounting for the change in specific heat of the condensate, and $c_{p,l}$ is the condensate specific heat.[4, 8]

An example of the dropwise and filmwise heat transfer coefficients as a function of condenser subcooling yielded from the above models is plotted in Figure S2 under typical experimental

conditions for condensation of water. The dropwise condensation heat transfer coefficient decreases at low subcooling because the interfacial heat transfer coefficient becomes a major resistance to heat transfer.[3b, 10] Meanwhile, the filmwise condensation heat transfer coefficient increases at low subcooling as the film becomes thinner at low heat fluxes[11] (note that, although the heat transfer coefficient is increasing, the heat flux is decreasing). Furthermore, the Nusselt theory used for filmwise condensation does not consider the interfacial heat transfer coefficient, which would decrease the heat transfer coefficient at low subcooling in competition with the increase due to the thinner film. This results in the dropwise and filmwise heat transfer coefficients approaching each other as subcooling decreases.

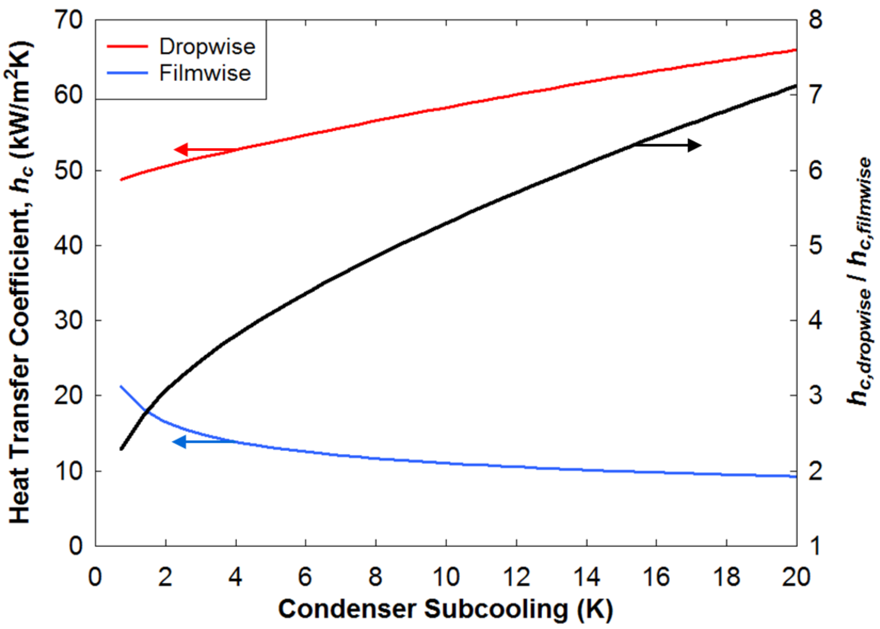

Figure S2. *Plot of model results for filmwise and dropwise condensation heat transfer coefficient as a function of condenser subcooling, keeping the condenser temperature constant at 15.5 ℃ (P_{sat} = 1780 Pa) and varying the surrounding saturated water vapor temperature from 16 to 35.5 ℃ (P_{sat} = 1840 to 5860 Pa). The secondary (right) vertical axis is the ratio of dropwise to filmwise condensation heat transfer coefficients.*

In the present study, the condenser subcooling during dropwise condensation of water ranged from ≈1.5-6 K. The experimentally determined heat transfer coefficient enhancement of 3-4x for dropwise condensation of water on flat hydrophobic and LIS-coated tubes compared to filmwise condensation is in excellent agreement with the model shown in Figure S2 in the 2-5 K subcooling range where the experimental measurements were taken. Note that, at higher subcooling over ≈10 K, the typically reported heat transfer coefficient enhancement of up to one order of magnitude[3b] for dropwise compared to filmwise condensation would be realized.

S7. Lubricant Layer Thickness

The lubricant layer thickness was approximated by dividing the total volume of lubricant added to the surface by the projected surface area of the condenser tube. The total volume of added lubricant was calculated with Equation S25 based on the procedure described in the main text:

$$V_{lub} = n\frac{4}{3}\pi {r_{lub}}^3 \tag{S25}$$

where n is the number of droplets of lubricant added to the surface (1 droplet in the present work) and r_{lub} is the radius of each droplet of lubricant added to the surface (1 mm in the present work). The surface area of the condenser tube is:

$$A_{cond} = 2\pi r_{tube} L_{tube} \tag{S26}$$

where r_{tube} and L_{tube} are the radius and the length of the condenser tube, respectively. Then, the thickness of the lubricant layer is:

$$t_{lub} = V_{lub}/A_{cond} \tag{S27}$$

In the present work, this lubricant layer thickness is 1.6 μm, on the same order as the copper oxide nanostructures, which have a characteristic height of 1 to 2 μm.[12] Therefore, the lubricant completely filled the nanostructured surface without significant excess. The thermal resistance

of the lubricant layer was also estimated to determine its effect on the overall heat transfer performance. The thermal resistance is:

$$R_{lub} = t_{lub}/k_{lub} \tag{S28}$$

where k_{lub} is the thermal conductivity of the lubricant, ≈ 0.09 W/m-K for Krytox GPL 101. Therefore, in the present work, the thermal resistance of the lubricant is approximately $1.8\text{x}10^{-5}$ m^2K/W. Comparing this to the thermal resistances for filmwise condensation of water ($8.2\text{x}10^{-5}$ m^2K/W) and toluene ($5.1\text{x}10^{-4}$ m^2K/W), it is clear that, even with the lubricant layer, we can expect a maximum heat transfer enhancement of up to 4.5x for water and 28x for toluene compared to filmwise condensation based on this simplified analysis. In fact, the actual maximum enhancement is greater due to the relatively high thermal conductivity of the copper oxide nanostructures compared to the lubricant, which essentially form a composite material with the lubricant and significantly lower the thermal resistance.

S8. Transition to Filmwise Condensation of Toluene

Although toluene has a finite positive contact angle on the flat hydrophobic condenser, the low value of contact angle and high contact angle hysteresis result in a transition to filmwise condensation even at very low subcooling of less than 1 K, shown in Figure S3 below. This phenomenon has been reported previously, where low-contact-angle fluids typically condense in the filmwise mode.[13]

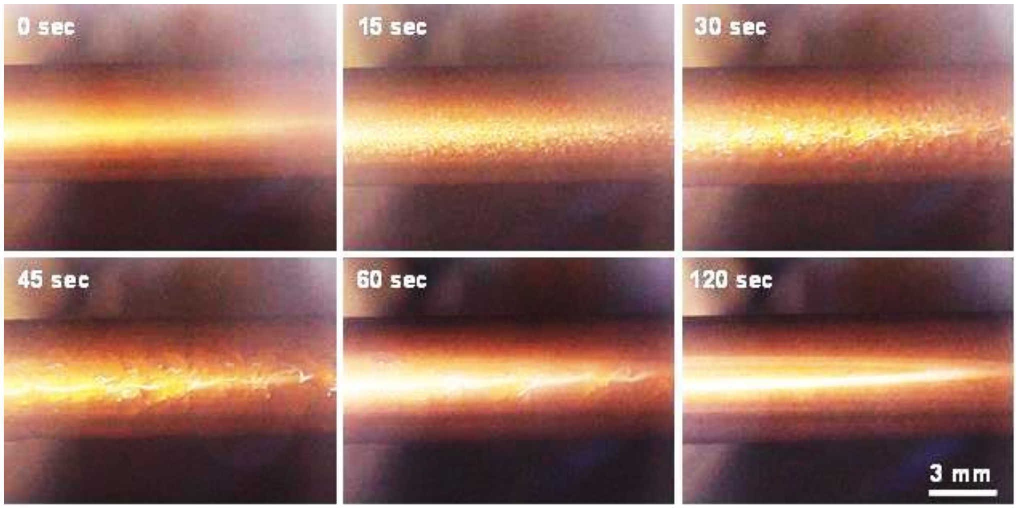

Figure S3. *Transition from dropwise to filmwise condensation for toluene condensing on the flat hydrophobic surface. These time-lapse images show that discrete droplets initially form and grow on the surface; however, due to the low contact angle and high contact angle hysteresis, the condensation mode transitions to filmwise even at low (<1 K) subcooling.*

SUPPLEMENTARY INFORMATION REFERENCES

1. Miljkovic, N.; Enright, R.; Nam, Y.; Lopez, K.; Dou, N.; Sack, J.; Wang, E. N., Jumping-Droplet-Enhanced Condensation on Scalable Superhydrophobic Nanostructured Surfaces. *Nano Lett* **2013,** *13* (1), 179-187.
2. Xiao, R.; Miljkovic, N.; Enright, R.; Wang, E. N., Immersion Condensation on Oil-Infused Heterogeneous Surfaces for Enhanced Heat Transfer. *Sci Rep-Uk* **2013,** *3*, 1988.
3. (a) Ma, X. H.; Zhou, X. D.; Lan, Z.; Li, Y. M.; Zhang, Y., Condensation Heat Transfer Enhancement in the Presence of Non-Condensable Gas Using the Interfacial Effect of Dropwise Condensation. *Int J Heat Mass Tran* **2008,** *51* (7-8), 1728-1737; (b) Rose, J. W., Dropwise condensation theory and experiment: a review. *P I Mech Eng a-J Pow* **2002,** *216* (A2), 115-128.
4. Incropera, F. P., *Introduction to heat transfer*. 5th ed.; Wiley: Hoboken, N.J., 2007; p 901.
5. Miljkovic, N.; Enright, R.; Wang, E. N., Modeling and Optimization of Superhydrophobic Condensation. *Journal of Heat Transfer* **2013,** *135* (11), 111004-111004.
6. Kaschiev, D., *Nucleation: Basic Theory With Applications*. Butterworth Heinemann: Oxford, 2000.
7. Rose, J. W.; Glicksman, L. R., Dropwise condensation **-** the distribution of drop sizes. *Int J Heat Mass Tran* **1973,** *16*, 411-425.
8. Carey, V. P., *Liquid-vapor phase-change phenomena : an introduction to the thermophysics of vaporization and condensation processes in heat transfer equipment*. 2nd ed.; Taylor and Francis: New York, 2008; p 742.
9. Weisensee, P. B.; Wanga, Y.; Hongliang, Q.; Schultz, D.; King, W. P.; Miljkovic, N., Condensate droplet size distribution on lubricant-infused surfaces. *Int J Heat Mass Tran* **2017,** *109*, 187-199.
10. (a) Wilmshurst, R.; Rose, J. W., Dropwise Condensation—Further Heat Transfer Measurements. In *Fourth International Heat Transfer Conference*, Elsevier: Paris-Versailles, France, 1970; Vol. 6; (b) Enright, R.; Miljkovic, N.; Alvarado, J. L.; Kim, K. J.; Rose, J. W., Dropwise Condensation on Micro- and Nanostructured Surfaces. *Nanoscale and Microscale Thermophysical Engineering* **2014,** *18* (3), 223-250.
11. Nusselt, W., The surface condensation of water vapour. *Z Ver Dtsch Ing* **1916,** *60*, 541-546.
12. (a) Preston, D. J.; Massachusetts Institute of Technology. Department of Mechanical Engineering., *Electrostatic charging of jumping droplets on superhydrophobic nanostructured surfaces : fundamental study and applications*. p 76 pages; (b) Preston, D. J.; Miljkovic, N.; Enright, R.; Wang, E. N., Jumping droplet electrostatic charging and effect on vapor drag. *Journal of Heat Transfer* **2014,** *136* (8), 080909; (c) Bhatia, B.; Preston, D. J.; Bierman, D. M.; Miljkovic, N.; Lenert, A.; Enright, R.; Nam, Y.; Lopez, K.; Dou, N.; Sack, J.; Chan, W. R.; Celanovic, I.; Soljacic, M.; Wang, E. N., Nanoengineered Surfaces for Thermal Energy Conversion. *15th International Conference on Micro and Nanotechnology for Power Generation and Energy Conversion Applications (Powermems 2015)* **2015,** *660,* 012036.
13. Cho, H. J.; Preston, D. J.; Zhu, Y.; Wang, E. N., Nanoengineering materials for liquid–vapour phase-change heat transfer. *Nature Materials Reviews* **2016,** *2*, 16092.
